# Granulosa cell insight: unraveling the potential of menstrual blood-derived stem cells and their exosomes on mitochondrial mechanisms in polycystic ovary syndrome (PCOS)

**DOI:** 10.1186/s13048-024-01484-3

**Published:** 2024-08-17

**Authors:** Mahna Mansoori, Somayeh Solhjoo, Maria Grazia Palmerini, Seyed Noureddin Nematollahi-Mahani, Massood Ezzatabadipour

**Affiliations:** 1 Anatomical Sciences Department, Afzalipour School of Medicine, Kerman University of Medical Sciences, Kerman, Iran; 2 Department of Life, Health and Environmental Sciences, University of L’Aquila, L’Aquila, Italy

**Keywords:** Polycystic ovary syndrome (PCOS), Granulosa cells, Menstrual blood-derived stem cells (MenSCs), Exosomes, Mitochondrial biogenesis

## Abstract

**Background:**

Polycystic ovary syndrome (PCOS) presents a significant challenge in women’s reproductive health, characterized by disrupted folliculogenesis and ovulatory dysfunction. Central to PCOS pathogenesis are granulosa cells, whose dysfunction contributes to aberrant steroid hormone production and oxidative stress. Mitochondrial dysfunction emerges as a key player, influencing cellular energetics, oxidative stress, and steroidogenesis. This study investigates the therapeutic potential of menstrual blood-derived stem cells (MenSCs) and their exosomes in mitigating mitochondrial dysfunction and oxidative stress in PCOS granulosa cells.

**Methods:**

Using a rat model of PCOS induced by letrozole, granulosa cells were harvested and cultured. MenSCs and their exosomes were employed to assess their effects on mitochondrial biogenesis, oxidative stress, and estrogen production in PCOS granulosa cells.

**Results:**

Results showed diminished mitochondrial biogenesis and increased oxidative stress in PCOS granulosa cells, alongside reduced estrogen production. Treatment with MenSCs and their exosomes demonstrated significant improvements in mitochondrial biogenesis, oxidative stress levels, and estrogen production in PCOS granulosa cells. Further analysis showed MenSCs' superior efficacy over exosomes, attributed to their sustained secretion of bioactive factors. Mechanistically, MenSCs and exosomes activated pathways related to mitochondrial biogenesis and antioxidative defense, highlighting their therapeutic potential for PCOS.

**Conclusions:**

This study offers insights into granulosa cells mitochondria’s role in PCOS pathogenesis and proposes MenSCs and exosomes as a potential strategy for mitigating mitochondrial dysfunction and oxidative stress in PCOS. Further research is needed to understand underlying mechanisms and validate clinical efficacy, presenting promising avenues for addressing PCOS complexity.

**Supplementary Information:**

The online version contains supplementary material available at 10.1186/s13048-024-01484-3.

## Introduction

Granulosa cells (GCs), a subgroup of steroidogenic cells within the ovarian follicles along with theca cells and follicular immune cells, play a pivotal role in the somatic cell population of ovarian follicles [1, 2]. Through close interaction with oocytes, these cells create a microenvironment for folliculogenesis and oocyte maturation [2, 3]. During this process, granulosa cells undergo numerous functional and differentiation changes, that lead them to maturity. These changes occur in response to factors secreted by the oocyte, ovarian factors, and endocrine factors. Notably, in response to follicle-stimulating hormone (FSH), granulosa cells express aromatase, converting androgens obtained from theca cells into estrogen. The synergistic action of estrogen with FSH induces the expression of luteinizing hormone receptor (LHR), This hormonal cascade facilitates granulosa cells maturation, the progression of folliculogenesis, ovulation, and optimal fertility [3, 4]. Disruption of the proper functioning of these cells can result in abnormal folliculogenesis and the disturbance of steroid hormones production, leading to pathological conditions such as granulosa cells tumors, premature ovarian failure (POF), and polycystic ovary syndrome (PCOS) [3, 5]. PCOS, in particular, emerges as a prevalent endocrine disorder that affects a substantial proportion of women of reproductive age, with estimates ranging from 6 to 21% [6, 7] in different populations. It represents a significant etiological factor in approximately 75% of cases involving anovulatory infertility [8]. Within the pathophysiology of PCOS lies a disruption in the normal follicular dynamics, resulting in a failure of dominant follicle selection and subsequent ovulation. This dysregulation appears histologically in the form of small and atretic follicles, characteristic of polycystic ovaries [4, 5, 9, 10].

Recent studies have emphasized the integral role of oxidative stress, endoplasmic reticulum (ER) involvement, aberrant apoptosis, and dysregulated autophagy in granulosa cells pathology, which significantly contribute to the clinical manifestations of PCOS [10, 11]. Central to cellular homeostasis, apoptosis regulation, and oxidative stress mitigation, mitochondria are pivotal organelles with multifaceted roles in cellular physiology and pathology [12, 13].

Mitochondrial dysfunction in PCOS results in oxidative stress, leading to granulosa cells apoptosis. the development of hyperandrogenism, insulin resistance, glucose intolerance, and follicular dysgenesis characteristic of PCOS [14, 15]. Mitochondrial anomalies in PCOS involve disruptions in mitochondrial biogenesis, including altered mitochondrial mass and number, changes in mitochondrial DNA copy number, and mutations in mitochondrial DNA [12, 16, 17]. These abnormalities collectively contribute to the complex pathophysiology of PCOS, emphasizing the crucial role of mitochondria in the syndrome’s development and clinical features [15, 18]. Amidst the backdrop of cellular dysfunction in PCOS, regenerative medicine emerges as a beacon of hope. This burgeoning field aims to regenerate and rejuvenate damaged and aging cells and tissues using cell-based and cell-free therapies [19].

Adult mesenchymal stem cells (MSCs), particularly menstrual blood-derived stem cells (MenSCs), have shown promise in tissue repair and enhancement of ovarian function [20]. MenSCs, which are harvested from a non-invasive and ethically uncomplicated source, have proven to be exceptionally effective in pre-clinical and clinical studies to repair cardiac and liver tissues, as well as to preserve ovarian reserves, in cases of POF [21–23].

The regenerative potential of MenSCs lies mainly in their secretions and paracrine mechanisms, including the secretion of extracellular vesicles such as exosomes [24, 25]. Exosomes, nano-sized vesicles released from cells, encapsulate molecules crucial for intercellular communication and immune regulation. The use of cell-secreted products, like exosomes, offers advantages including reduced tumorigenic potential and immunogenicity [19, 26].

Despite the critical role of granulosa cells mitochondria in ovarian function and the potential of MenSCs and their exosomes in regenerative therapies, research investigating their interaction is limited.

Therefore, this study aims to investigate the in vitro impact of MenSCs and their exosomes on granulosa cells derived from PCOS animal models, with a specific focus on mitochondrial characteristics. By elucidating the effects of these novel stem cell sources as well as their exosomes, we aim to help develop targeted therapies for PCOS patients.

## Materials and methods

### Induction and confirmation of polycystic ovary syndrome (PCOS) in rats

The present study was approved by the ethics committee of Kerman University of Medical Sciences, Kerman, Iran (approval number: IR.KMU.AH.REC.1400.138). Adult female Wistar rats aged 6–8 weeks were obtained from the animal farm of Kerman University of Medical Sciences. Rats were housed under standard conditions with free access to tap water and rodent food and controlled temperature (21 ± 3°C) and light (12 h light/12 h dark) and their estrous cycle was checked daily. Eight rats with at least two regular estrous cycles were chosen for next steps.

To induce PCOS, we followed a previously established protocol [27]. Briefly, (*n* = 5) we administered 1 mg/kg/day letrozole (Aburaihan Pharmaceutical Co, Iran) dissolved in 1% carboxymethyl cellulose (CMC) in distilled water through oral gavage for 21 days. The remaining three rats received 1% CMC for 21 days and served as the control group.

PCOS was confirmed in animals by observing irregular estrous cycles and examining ovarian cysts in histological sections stained with Mason trichrome.

### Extraction and culture *of granulosa* cells

Granulosa cells were extracted from control and PCOS-induced rats (PCOS-GCs). Rats were superovulated by injecting 60 IU of Pregnant Mare Serum Gonadotropin (PMSG) subcutaneously, and after 48 h, ovaries were removed under anesthesia by intraperitoneal injection of ketamine (40 mg/kg) and xylazine (5 mg/ kg). Ovaries were immediately washed with PBS and placed in DMEM/F12 medium (BioIdea, Iran). Follicles were punctured using 25-gauge insulin needles under a stereo microscope (Nikon, SMZ645, Japan), and GCs were collected using a sterile Pasteur pipette. After two washes, cells were transferred to a culture plate containing DMEM/F12, 10% FBS (Ana Cell tec., Iran), 100 mg/mL streptomycin, and 100 U/mL penicillin (DNAbiotech, Iran). Cells were incubated at 37°C with 5% carbon dioxide.

### Identification *of granulosa* cells characteristics

Follicle-stimulating hormone receptor (FSHR) expression was studied by immunocytochemical (ICC) analysis. 2 × 10^4^ GCs were seeded in 24-well plates for 72 h, fixed with 4% paraformaldehyde, and washed with ice-cold PBST. To block nonspecific binding sites, cells were incubated with 1% BSA and glycine in PBST. The primary antibody, rabbit polyclonal anti-FSH-R antibody (1:200 dilution; FSH-R; H-190: sc-13935 Santa Cruz Biotechnology), was applied overnight at 4°C. Cells were then incubated with FITC-conjugated goat anti-rabbit IgG secondary antibody (1:100 dilution; cat. E-AB-1014, Elabscience) for 1 h at room temperature, followed by 3 PBS washes. Cell nuclei were stained with DAPI for 1 min. FSHR expression was examined using a fluorescent microscope (Olympus BX50, Tokyo, Japan) with a blue filter and photographed by a digital camera.

### Isolation and cultivation of MenSCs

Menstrual Stem Cells (MenSCs) were harvested following a previously published protocol [27]. Briefly, four frozen vials of MenSCs, obtained from menstrual blood samples of healthy Iranian women aged 20–30 years with regular menstrual cycles and no apparent symptoms of Polycystic Ovary Syndrome, and confirmed through their mesenchymal markers, were thawed at room temperature, and passaged. 6 × 10^6^ cells were cultured in DMEM/F12 medium supplemented with 10% FBS, 100 mg/ml streptomycin, and 100 U/ml penicillin under standard conditions at 37°C with 5% carbon dioxide. The culture medium was replaced every three days. At 80–90%, confluence, MenSCs were detached from the substratum using trypsin and EDTA solution and re-cultured in culture dishes.

### Isolation and identification of MenSC-derived exosomes (MenSC-EXO)

MenSCs were cultured until they reached 75% confluence. Subsequently, the medium was aspirated, and the cells were washed three times with sterile PBS. The cells were then cultured in fresh serum-free culture medium for 48 h at 37 °C with 5% CO2 and the conditioned medium was collected, followed by a series of centrifugation steps (300 × g for 10 min, 2000 × g for 20 min and 10,000 × g for 30 min) to eliminate dead cells and debris.

Exosomes were isolated through high-speed centrifugation at 100,000 × g for 70 min at 4 °C (Vision VS-30000i, Korea). The resulting pellet was subjected to a second high-speed centrifugation and the resulting exosome pellet was suspended in 100 ul of PBS. The isolated exosomes were stored at -80 °C until further use.

Exosomes characteristics including microstructure, dynamic light scattering (DLS) for exosome diameters, and specific exosome markers CD63 and CD9 were determined by transmission electron microscopy (EM 208S Philips, Netherlands), DLS (VASCO2, Cordouan Technology, France) and Western blotting using anti-CD63 (diluted 1:500) (MX-49.129.5: sc-5275, Santa Cruz Biotechnology), anti-CD9 (diluted 1:500) (C-4: sc-13118, Santa Cruz Biotechnology) and secondary antibodies (m-IgGκBP-HRP: sc-516102, mouse anti-rabbit IgG-HRP: sc-2357), respectively.

### Protein quantification

Exosome samples suspended in PBS were lysed using RIPA Buffer (Anacell, Iran) for 15 min on ice with occasional vortexing. Protein concentration was then quantified using a bicinchoninic acid (BCA) assay with the Santa Cruz BCA Protein Assay kit (Santa Cruz Biotechnology, Santa Cruz, CA) according to the manufacturer’s instructions.

### Uptake of PKH67-labeled MenSCs-EXO by GCs

Exosome uptake by granulosa cells was assessed using PKH67 fluorescent dye kit as recommended by manufacturer (Sigma Aldrich, Mo, USA). The exosome solution was mixed with diluent C and then stained with diluted PKH67 ethanolic dye. After a 5-min incubation, the reaction was stopped with FBS. Labeled exosomes were added to the culture medium of each well of a 6-well plate containing 2 × 10^5^ granulosa cells, incubated for 24 h, and then washed with PBS. Cells were then fixed with paraformaldehyde and stained with Hoechst 33,258 Dye Solution (ab228550, UK). A fluorescent microscope (Olympus 1X71, Tokyo, Japan) was used to examine cells and visualize the labeled exosomes.

### MTT assay

The MTT assay determined the optimal dose of MenSCs-EXO treatment. Initially, 1 × 10^4^ GCs from PCOS-induced rats were seeded in a 96-well plate and treated with various concentrations of MenSCs-EXO (0, 4, 8, 16, 20, 50, 100 μg/mL). After 24 h of incubation, the cell culture medium was removed, and the cells were washed with PBS. Then, 100 μl of MTT solution (5 mg/ml in DMEM) was added to each well, followed by four hours of incubation at 37°C. After discarding the supernatant, 100 μl of DMSO was added to each well and incubated for an hour in the dark with shaking. Absorbance was measured at 570 nm using a microplate reader (Biotek, USA), and cell viability was calculated as the ratio of absorbance in the treated group to untreated group.

### Experimental groups

Granulosa cells extracted from both control and PCOS group were categorized into distinct subgroups for further investigation. These subgroups were: **Control**, consisting of granulosa cells obtained from control rats without any interventions; **PCOS**, comprising granulosa cells derived from PCOS-induced rats without any interventions; **MenSCs**, in which granulosa cells derived from PCOS-induced rats were co-cultured with MenSCs at a ratio of 5 to 1; and **MenSCs-Exo**, in which granulosa cells from PCOS-induced rats were treated with exosomes derived from MenSCs at a concentration of 8 ug/mL.

For each subgroup, 2 × 10^5^ granulosa cells were seeded per well in a 6-well plate. After 24 h of incubation at 37°C with 5% CO2, the cells were examined according to the study design.

### Assessment of MDA level

MDA levels were measured using thiobarbituric acid (TBA) in culture media. Samples were mixed with trichloroacetic acid and thiobarbituric acid, boiled, cooled, and centrifuged. The absorbance of the pink phase was measured at 534 nm. MDA concentrations (nmol/mL) were determined using a standard curve.

### Assessment of superoxide dismutase (SOD) activity

SOD activity was measured using a biochemical kit from Navand Salamat Co., Iran, based on pyrogallol autoxidation. Cells were lysed, and supernatants were collected for assay. Absorbance at 405 nm was measured after incubation with assay solutions and the SOD activity was calculated using the following formula.

$$\frac{\text{OD of }tests}{\text{OD of control}}\times 200$$

### Assessment of estradiol level

To measure estradiol concentrations, conditioned media from each experimental group were collected after 24 h of treatment. An ELISA kit from DiaMetra, Italy, was used for quantification. Briefly, 25 μl of standards and samples were added in duplicate to microwell plates. Then 200 μl of 17β estradiol-HRP conjugate was added to each well and incubated for 2 h at 37°C. After washing the microplate, 100 μl of TMB substrate was added to each well and incubated for 30 min at room temperature in the dark. Finally, 100 μl of stop solution was added, mixed, and absorbance was recorded at 450 nm using a microplate reader (Biotek, USA).

### Assessment of pgc-1α gene expression:

To evaluate the mitochondrial biogenesis of GCs, the expression of PGC-1α gene was examined by Real-Time PCR. Following 24 h of treatment, the culture medium was removed, 5 × 10^5^ cells were washed with PBS, and lysed using TRIzol (Yektatazhiz, Iran). RNA was extracted using the chloroform and isopropanol methods. RNA quality and quantity were assessed using a NanoDrop spectrophotometer (Thermo Fisher Scientific, Wilmington, United States). Subsequently, cDNA was synthesized using a reverse transcription kit (Parstous, Iran). Primers were designed as shown in Table 1.

**Table 1:** Primer sequences for PCR analysis

Primer	Forward sequence	Reverse sequence
PGC1α	5’-GTGCAGCCAAGACTCTGTATGG-3’	5’-GTCCAGGTCATTCACATCAAGTTC-3’
GAPDH	5’-AAGTTCAACGGCACAGTCAAGG-3’	5’-CATACTCAGCACCAGCATCACC-3’
mt-DNA	5’-TAGCCATCCCCCTATGAGCA-3’	5’-CTTGCGGTAAGAAGTGGGCT-3’
n-DNA	5’-AAGTTCAACGGCACAGTCAAGG-3’	5’-CATACTCAGCACCAGCATCACC-3’

For quantitative real-time PCR (qRT-PCR), SYBR Green Master Mix (Amplicon, Denmark) was used along with cDNA and gene-specific primers. The Light Cycler Real-Time PCR System (MIC, Queensland, Australia) was used for PCR analysis with the following cycling conditions: initial denaturation at 95°C for 5 min, 40 cycles at 95°C for 23 s for denaturation, followed by annealing at 60°C for 30 s, and extension at 72°C for 30 s. Relative gene expressions were determined and normalized to GAPDH expression, using the 2 − ΔΔCT method.

### Assessment of mitochondrial copy number

In this study, DNA extraction from GCs involved trypsinization, washing, and overnight incubation with lysis buffer and proteinase K. DNA was then separated using the phenol–chloroform method, followed by precipitation, and washing with ethanol, sodium citrate, and 70% ethanol, respectively. The dried DNA pellet was suspended in distilled water. Concentration and purity were assessed using a Nanodrop spectrophotometer.

The extracted DNA was used to examine mitochondrial DNA (mtDNA) copy numbers relative to nuclear DNA (nDNA) using Real-Time PCR, and the 2-ΔΔCT method. qRT-PCR was performed by combining DNA samples with specific primers for mtDNA and nDNA (Table 1), along with a master mix. The qRT-PCR procedure involved initial denaturation at 95°C for 5 min, followed by 45 cycles of denaturation at 95°C for 10 s, annealing at 60°C for 10 s, and extension at 72°C for 20 s.

### Assessment of PGC-1α by immunofluorescence staining

GCs (1 × 10^5^) were seeded in 12-well plates and treated with MenSCs and MenSCs-EXO for 24 h to evaluate PGC-1α using ICC. The cells were washed with PBS, fixed with 4% paraformaldehyde, permeabilized with 1% Triton X-100 in PBS, and blocked with 1% BSA in TBS.

The cells were incubated with rat Anti- PGC-1α antibody (diluted 1:300; cat.no. ab54481, Abcam, US) for 4 h, washed with PBS, and incubated with FITC-conjugated Goat Anti-Rabbit IgG secondary antibody (diluted 1:100; cat.no. E-AB-1014, Elabscience, US) for 1 h at room temperature. Cells nuclei were stained with DAPI and the.

slides were observed using a fluorescence microscope (Nikon, Japan) at 400 × magnification. Image intensity analysis was performed using ImageJ Fiji software (version 1.52; WS Rasband, National Institute of Health, Bethesda, Rockville, MD, US) following a validated protocol [28].

### Statistical analysis

All data were analyzed using Graph pad Prism 8 software. One-sample Kolmogorov–Smirnov test was used to check the normality of the data distribution, then the data were analyzed with One-way Anova and Post Hoc Tukey test. Statistically significance was considered at p ≤ 0.05.

## Results

### PCOS model confirmation

**Fig. 1:**
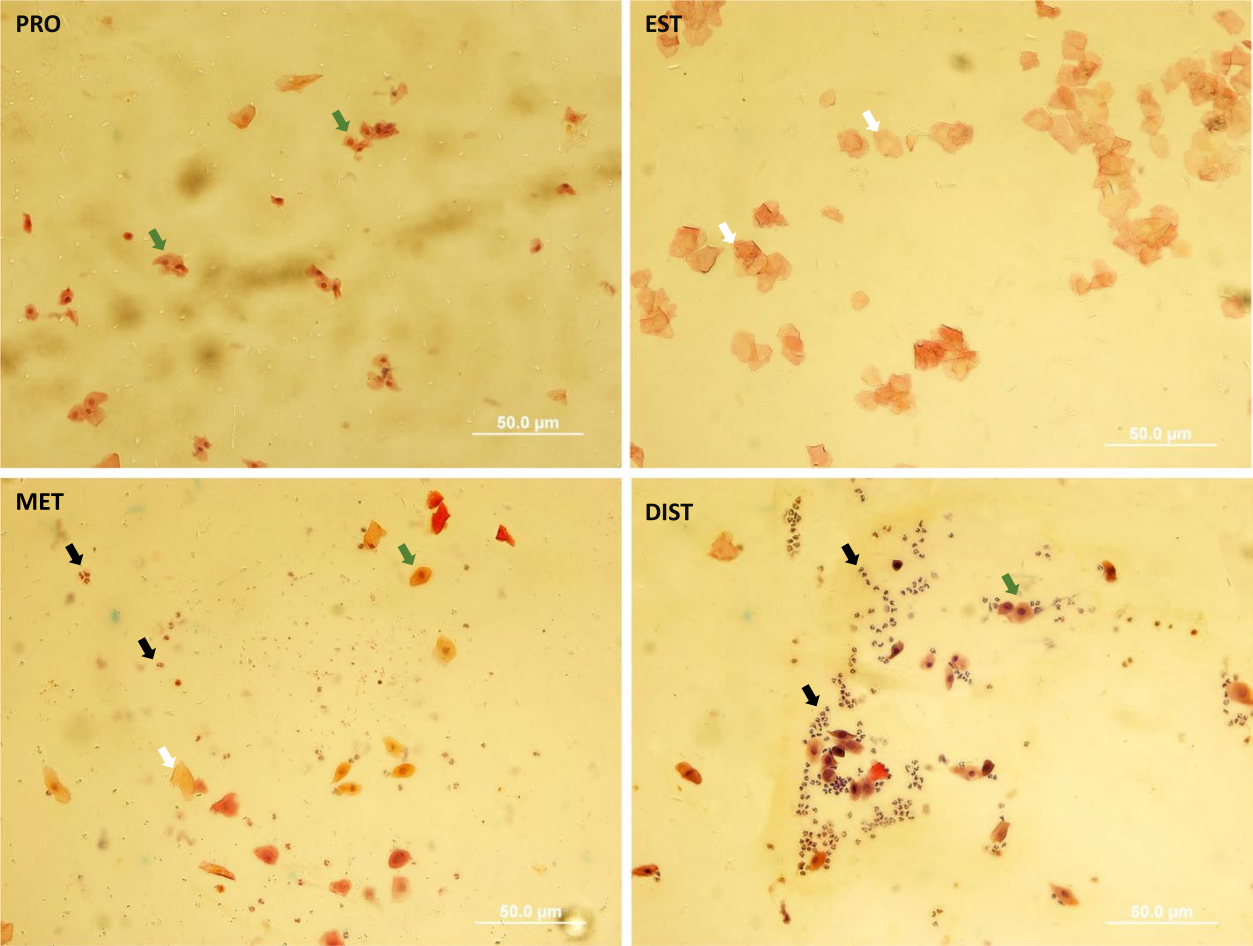
Photomicrographs illustrate the characteristic cytology of vaginal smears obtained from control rats. In rats, the cycle stages were classified as proestrus (PRO), characterized by a predominant population of nucleated epithelial cells (green arrow); estrus (EST), distinguished by cornified cells (white arrow); metestrus (MET), identified by the presence of both cornified epithelial cells (white arrow), nucleated epithelial cells (green arrow) and leukocytes (black arrow); and the diestrus stage (DIST), where leukocytes predominate alongside nucleated epithelial cells (green arrow).” 400 × magnification; bars = 50 μm

**Fig. 2:**
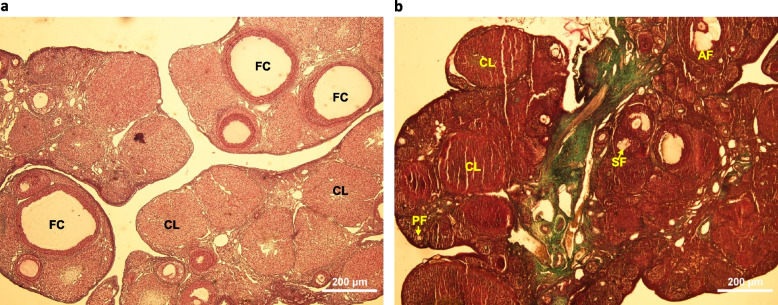
Histological photomicrographs of rat ovaries stained with Mason trichrome. **a** PCOS group; Displays the presence of follicular cysts (FC) and the absence of normal follicle development. **b** Control group; Shows normal ovarian structures, including Corpus luteum (CL), Primary Follicle (PF), Secondary Follicle (SF), and Antral Follicle (AF).; 40 × magnification; bars = 200 μm

The polycystic ovary syndrome (PCOS) model was confirmed by observing at least 2 consecutive irregular estrous cycles (Fig. 1) and multiple ovarian cysts in histological sections of sample PCOS-induced animals compared with the control group (Fig. 2 a, b).

### Granulosa cells culture and confirmation

**Fig. 3:**
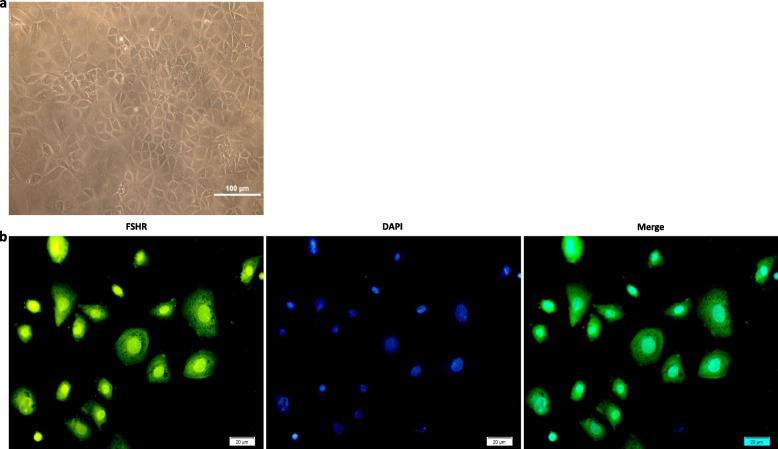
Morphological characteristic and Follicle-stimulating hormone receptor (FSHR) expression assay of granulosa cells In-vitro. **a** The morphological characteristics of granulosa cells were visualized using an inverted microscope. The cells exhibited an epithelial-like morphology, with a flat and polygonal shape. Nuclei appeared round. 100 × magnification; bars = 100 μm. **b** Immunofluorescence staining of FSHR (green) was performed in granulosa cells obtain from rat ovaries. The nuclei were stain with DAPI (blue). 400 × magnification; bars = 20 μm

Following PCOS model validation, granulosa cells were extracted from rat ovaries and cultured. The harvested cells exhibited flat and polygonal cells, resembling epithelial cells. Their nuclei appeared to be round, and the cytoplasm contained several granules (Fig. 3a).

ICC analysis confirmed expression of the specific membrane receptor for granulosa cells, follicle-stimulating hormone receptor (FSHR) (Fig. 3b).

### Characteristics and the uptake of menstrual blood-derived mesenchymal stem cell exosomes by* Granulosa* cells

**Fig. 4:**
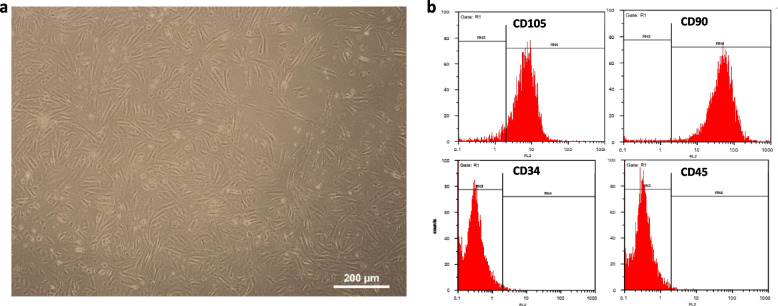
Morphological characteristic and identification of Menstrual Blood-Derived Mesenchymal Stem Cells (MenSCs). **a** Morphology of passage 4–6 MenSCs observed under an inverted microscope showing spindle-shaped cells. 40 × magnification; bars = 200 μm. **b** Surface marker expression profile of MenSCs analyzed by flow cytometry; these cells were positive for CD105, CD90, and negative for CD34 and CD45, confirming their mesenchymal stem cell identity

Exosomes were isolated from a serum-free culture medium using passages 4 to 6 MenSCs after 48 h of cultivation (Fig. 4a). These cells had been previously confirmed and employed in the ongoing study [27] (Fig. 4b).

**Fig. 5:**
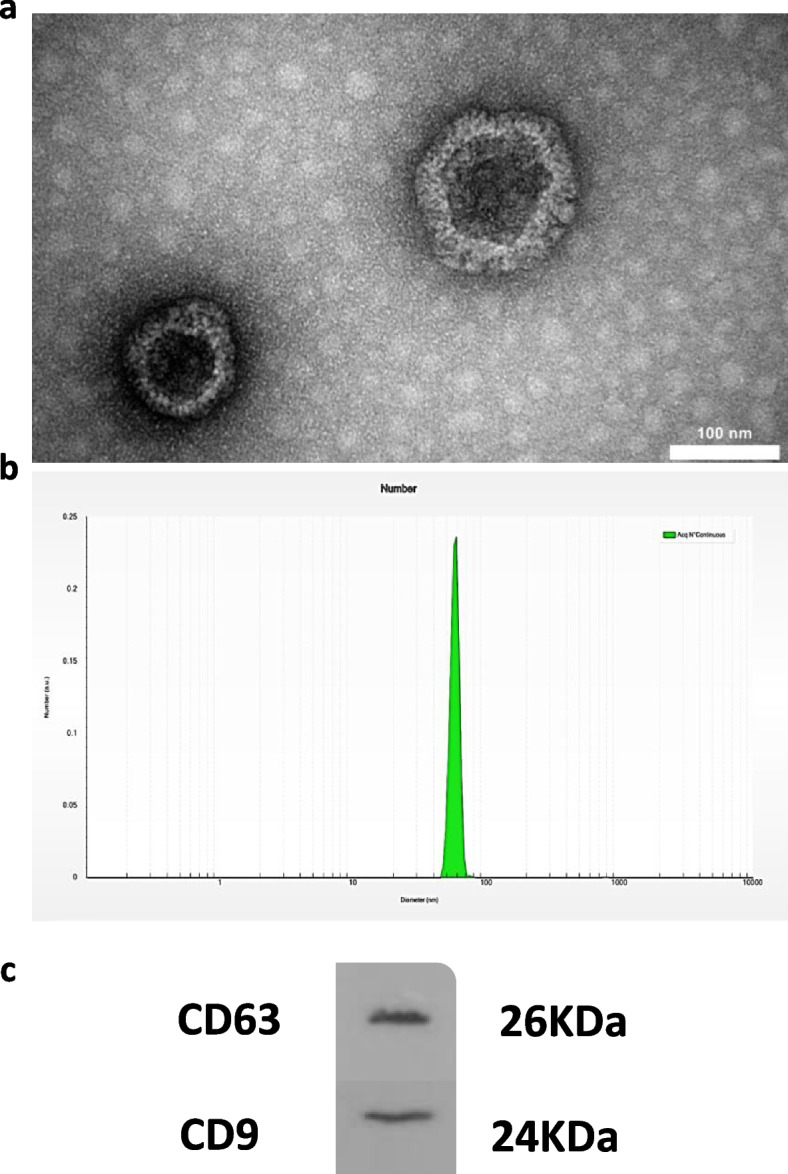
Characterization of exosomes derived from Menstrual Blood-Derived Mesenchymal Stem Cells (MenSCs-EXO). **a** Morphology of MenSCs-EXO observed under a transmission electron microscope demonstrating a heterogenous size mixture of cup-shaped vesicles ranging from 50–150 nm. **b** Particle diameter distribution range of MenSCs-EXO measured by Dynamic Light Scattering (DLS) in triplicate revealed an average diameter of 59 nm. **c** Western blot analysis demonstrated the presence of exosome markers CD63 and CD9 in MenSCs-EXO

Transmission electron microscopic evaluation of MenSCs-EXO particles revealed a cup-shaped structure within a size range of 50–150 nm, consistent with Dynamic Light Scattering (DLS) results. The DLS analysis indicated a mean particle size of 59 ± 4.8 nm. Additionally, Western blot analysis demonstrated that MenSCs-EXO were enriched with exosomal markers CD63 and CD9 (Fig. 5a-c).

**Fig. 6:**
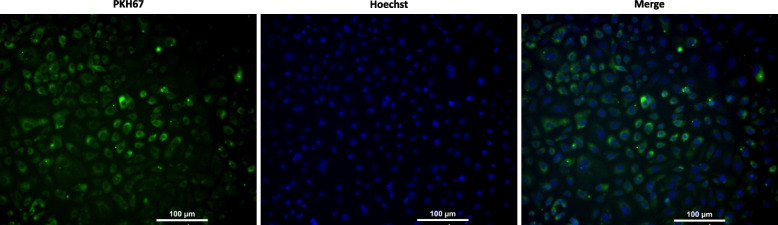
Uptake of PKH67-labeled MenSCs-EXO by granulosa cells In-vitro. Exosomes, purified from condition media of MenSCs culture, were labeled with PKH67 dye and co-cultured with primary culture of granulosa cells for 24 h. Green fluorescence indicates the successful uptake of PKH67-labeled MenSCs-EXO by the granulosa cells, while blue fluorescence represents nuclear staining with Hoechst. 100 × magnification; bars = 100 μm

To explore the uptake of MenSCs-EXO by granulosa cells, the particles were labeled with PKH67 and co-incubated with granulosa cells. After 24 h of incubation, the granulosa cells showed successful uptake of MenSCs-EXO-labeled with PKH67 membrane staining dye (Fig. 6).

### Cell viability assay

**Fig. 7:**
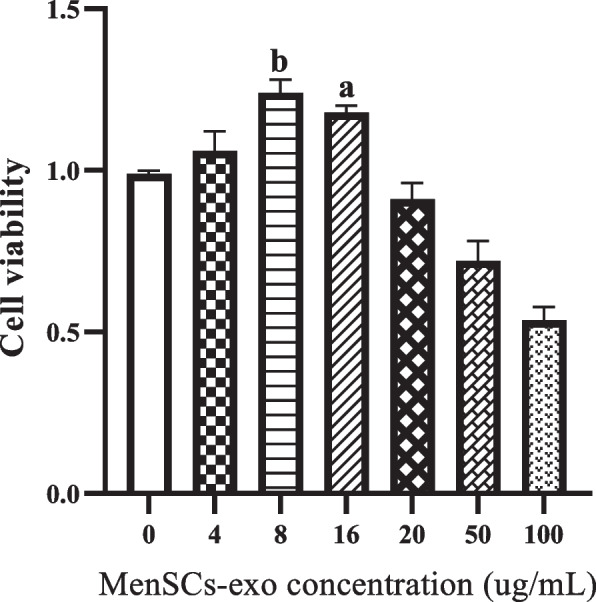
Graph of MTT Viability Assay Conducted on PCOS Granulosa Cells Exposed to Different Concentrations of MenSCs-Exo Over a 24-h Period. The concentrations of 8 and 16 μg/mL MenSCs-exo significantly increased proliferation compared to non-treated cells. Data are presented as mean ± standard deviation. *a* = *P* ≤ *0.*0006 vs non-treated cells and *b* = *P* ≤ *0.0001* vs non-treated cells

MTT assay was conducted to evaluate the viability of PCOS-GCs after treatment with different concentrations of MenSCs-EXO. The results revealed that at a concentration of 8 ug/mL the viability of the treated cells was similar to non-treated cells and significantly higher compared to other concentrations (*P* = 0.0001 vs non-treated cells.). As a result, this particular dose was chosen for subsequent experimentation (Fig. 7).

### Partial modulation of oxidative stress in PCOS model through MenSCs and MenSCs-EXO treatments

Our findings showed that PCOS-GCs exhibit significantly elevated levels of MDA compared to the GCs (*P* = 0.003). In the PCOS-GCs group, the mean MDA level was measured as 1.722 ± 0.745 nmol/ml, whereas in the control group it was 0.702 ± 0.1219 nmol/ml, suggesting oxidative stress conditions.

**Fig. 8:**
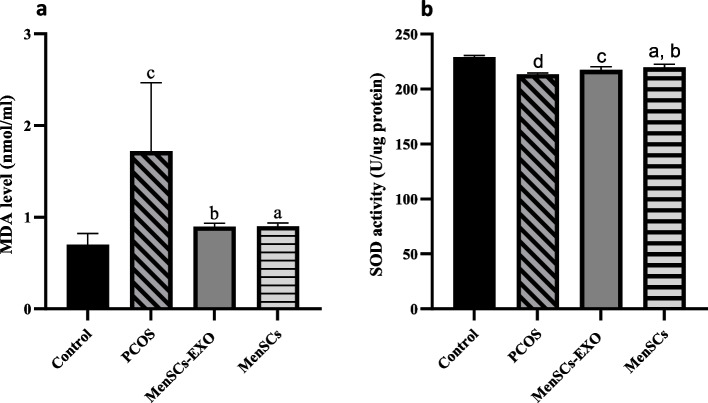
Oxidative Stress Parameters in Granulosa Cells following treatment with MenSCs and MenSCs- EXO. **a** MDA concentrations in culture medium of different groups showing higher levels in PCOS compared to control, with reductions following treatment with MenSCs and MenSCs-EXO. Data are presented as mean ± standard deviation. *a* = *P* ≤ *0.016* vs PCOS; *b* = *P* ≤ *0.015* vs PCOS; *c* = *P* ≤ *0.003* vs Control. **b** SOD Activity in cell lysates, showing a significant increase in MenSCs-treated cells compared to PCOS, while MenSCs-EXO treatment did not significantly change SOD activity. Data are presented as mean ± standard deviation. *a* = *P* ≤ *0.011* vs PCOS; b = *P* ≤ *0.001* vs Control; *c* = *P* ≤ *0.002* vs Control and *d* = *P* ≤ *0.0001* vs Control

However, treatment of PCOS-GCs with MenSCs and MenSCs-EXO displayed a notable reduction in MDA levels (*P* = 0.016 and *P* = 0.015 vs PCOS-GCs), with values reaching 0.902 ± 0.034 nmol/ml and 0.898 ± 0.034 nmol/ml, respectively. No significant difference was observed between the PCOS-GCs treated with MenSCs and MenSCs-EXO groups (Fig. 8a).

In addition to the change in MDA levels, treatment of PCOS-GCs with MenSCs led to significant activity of SOD. However, treatment of PCOS-GCs with MenSCs-Exosomes did not yield a statistically significant increase in SOD activity (Fig. 8b).

### Enhanced steroidogenic activity of PCOS-GCs treated with MenSCs and MenSCs-EXO

**Fig. 9:**
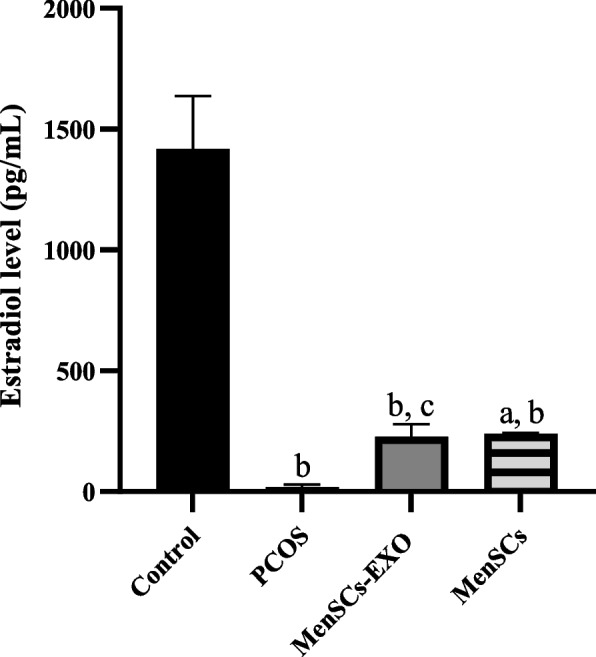
Estradiol level in culture medium of different groups following treatment with MenSCs-EXO and MenSCs*.* Estradiol levels were significantly lower in PCOS compared to control. Treatment with both MenSCs and MenSCs-EXO led to a substantial increase in estradiol production in PCOS granulosa cells. Data are presented as mean ± standard deviation. *a* = *P* ≤ *0.007* vs PCOS; *b* = *P* ≤ *0.0001* vs Control + PCOS and *c* = *P* ≤ *0.012* vs PCOS

The steroidogenic activity in granulosa cells of different groups was evaluated by measuring the concentration of estradiol in the conditioned media after the completion of the treatment period. After 24 h, the level of estradiol in the control group (GCs) was 1419 ± 218.1 pg/ml, which was significantly (*P* = 0.0001) less than the value (30.8 pg/ml) in the PCOS-GCs. Treatment of PCOS-GCs with both MenSCs and MenSCs-EXO led to an approximately 12-fold increase in the concentration of estrogen produced (226.9 ± 51.8 pg/ml and 239.6 ± 2.2 pg/ml, respectively, *P* = 0.007 and *P* = 0.012 compared with PCOS-GCs), (Fig. 9).

### Enhanced mitochondrial biogenesis in PCOS-GCs treated with MenSCs and MenSCs-EXO

**Fig. 10:**
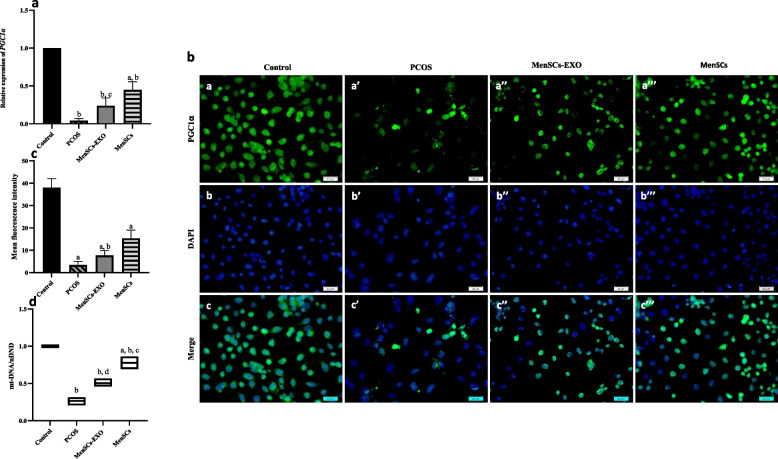
Mitochondrial Biogenesis Status. **a** Relative Expression Level of Gene PGC1α by real time PCR. Results were normalized at first with GAPDH and then to the Control. Data are presented as mean ± standard deviation. *a* = *P* ≤ *0.002* vs MenSCs-EXO + PCOS + Control; *b* = *P* ≤ *0.0001* vs MenSCs-EXO + PCOS + Control and *c* = *P* ≤ *0.004* vs PCOS. **b** Immunofluorescence Staining of PGC1α (green) was performed in granulosa cells. The nuclei were stain with DAPI (blue). 400 × magnification; bars = 20 μm. **c** Semi-quantification of Mean Fluorescence Intensity of PGC1α in granulosa Cells following treatment with MenSCs-EXO and MenSCs. Data are presented as mean ± standard deviation. *a* = *P* ≤ *0.0001* vs MenSCs-EXO + PCOS + Control; *b* = *P* ≤ *0.044* vs PCOS. **d** Relative mtDNA Copy Number in Granulosa Cells of different groups were measured by quantitative real-time PCR (qPCR) and reported as a ratio of mitochondrial DNA (mt-DNA) to the nuclear DNA (nDNA). Data are presented as mean. *a* = *P* ≤ *0.001* vs MenSCs-EXO; *b* = *P* ≤ *0.0001* vs PCOS + Control and *c* = *P* ≤ *0.006* vs Control and *d* = *P* ≤ *0.004* vs PCOS

To investigate mitochondrial biogenesis, we evaluated the expression of the PGC-1α factor at both gene and protein levels using qTR-PCR and ICC. Our findings revealed a significant (*P* = 0.0001) decrease in PGC-1 α gene expression in PCOS-GCs compared to those from the GCs group, indicating impaired mitochondrial biogenesis in PCOS. Interestingly, treatment with MenSCs and MenSCs-EXO resulted in a significant (*P* = 0.0001and *P* = 0.004 vs PCOS-GCs) increase of PGC-1α expression (Fig. 10a), ICC analysis further supported gene expression results, demonstrating a marked increase in PGC1α protein levels following treatment of PCOS-GCs with MenSCs and MenSCs-EXO (Fig. 10b).

Notably, the mean fluorescence intensity of PGC-1α protein in the GC of control group was significantly (*P* = 0.0001) higher than that in the PCOS-GCs group. Treatment of PCOS-GCs with MenSCs exhibited a nearly two-fold increase in PGC-1α protein expression compared to MenSCs-EXO (*P* = 0.0001 vs MenSCs-EXO), indicating the superior efficacy of cell-based therapy in restoring mitochondrial biogenesis (Fig. 10c).

Another mitochondrial biogenesis factor is relative mitochondrial DNA content, which in this study was assessed using qRT-PCR to determine the mtDNA to nuclear DNA ratio in granulosa cells from different groups. Our data revealed a significantly (P = 0.0001) higher mtDNA content in the GCs of control group compared to the PCOS-GCs group. Additionally, treatment of PCOS-GCs with both MenSCs and MenSCs-EXO led to a significant (*P* = 0.0001and *P* = 0.004, respectively) increase in the relative mtDNA content compared to the PCOS-GCs group. Specifically, this ratio reached 0.5042 ± 0.055 in the MenSCs-EXO group and 0.7766 ± 0.084 in the MenSCs group, while it was 0.2662 ± 0.054 in the PCOS-GCs group (Fig. 10d). Furthermore, a significant difference was observed between the two treatment groups (*P* = 0.001 vs MenSCs-EXO).

## Discussion

Polycystic ovary syndrome (PCOS) stands as a prevalent endocrine disorder affecting women of reproductive age and is recognized as a primary metabolic concern in developing countries. This condition disrupts normal folliculogenesis and often leads to ovulation disorders and, as a result, infertility [4, 5, 7].

In the complex process of folliculogenesis, granulosa cells emerge as pivotal contributors, orchestrating the production of crucial growth factors such as insulin-like growth factor (IGF) and steroid hormones [29, 30]. Although the etiology of PCOS remains incompletely elucidated, mounting evidence implicates metabolic perturbations along with aberrant proliferation, differentiation, and apoptosis of granulosa cells in its pathogenesis [1, 10, 16, 31]. The present investigation squarely targets granulosa cells as central players in the progression of PCOS. Our findings emphasized diminished mitochondrial function, decreased estrogen synthesis, and increased oxidative stress in granulosa cells harvested from PCOS-induced rats. Moreover, this study unveiled the therapeutic potential of menstrual blood-derived stem cells (MenSCs) and their exosomes in modulating mitochondrial biogenesis and oxidative stress in PCOS model granulosa cells. Through in vitro experimentation, we elucidated the beneficial impacts of MenSCs and their exosomes for mitigating mitochondrial dysfunction and oxidative stress in PCOS. Given the pivotal role of mitochondria in cellular energetics and fate determination, elucidating mitochondrial dysfunction and its contributing factors is of great importance in understanding the pathogenesis of PCOS.

Mitochondria play a vital role in regulating both the normal and pathological functions of granulosa cells, essential for cellular health, oocyte quality, and embryonic development [13, 32]. Dysfunctions in granulosa cells mitochondria are implicated in various ovarian disorders and infertility, including aging [33], endometriosis [34], premature ovarian insufficiency (POI) [35], and PCOS [18].

Mitochondrial dysfunction encompasses disturbances in mitochondrial biogenesis, a process critical for generating new mitochondria and maintaining cellular health. The master regulator of mitochondrial biogenesis, PGC-1α, orchestrates the activation of genes necessary for mitochondrial preservation, replication, and translation of mitochondrial DNA (mtDNA), vital for the respiratory chain [36, 37].

Our investigation demonstrated a significant decrease in mtDNA copy number and PGC-1α expression in PCOS granulosa cells compared to controls, indicating impaired mitochondrial biogenesis in PCOS.

We explored the therapeutic potential of MenSCs in PCOS by co-culturing with granulosa cells and treating them with MenSC-EXO. Treatment resulted in increased expression of PGC-1α and elevated mtDNA copy numbers, indicating improved mitochondrial biogenesis in PCOS granulosa cells.

Several studies support our current findings regarding impaired mitochondrial biogenesis in granulosa cells from PCOS patients. Xie et al. and Zhao et al. observed decreased mRNA expression of mitochondrial biogenesis factors such as PGC-1α in granulosa cells of PCOS patients compared to healthy individuals. Additionally, decreased mtDNA copy numbers were noted in PCOS granulosa cells, indicating compromised mitochondrial function [38, 39].

Similarly, research on PCOS model mice showed that the mRNA expression of mitochondrial biogenesis genes such as PGC-1α and NRF-1 was decreased in granulosa cells compared to the control group, which further supports the association between PCOS and impaired mitochondrial biogenesis [10].

However, Min et al. reported contrasting results, where induced pluripotent stem cells (iPSCs) derived from PCOS patients exhibited higher mtDNA copy numbers and enhanced mitochondrial biogenesis compared to those from healthy individuals. This discrepancy may stem from differences in disease progression and adaptive mechanisms in different cell types. During the early and mild stages of PCOS, cells may compensate for mitochondrial dysfunction by increasing mitochondrial biogenesis and mtDNA copy numbers. Notably, iPSCs inherently possess a greater metabolic capacity compared to somatic cells, which could contribute to their observed differences [15, 40].

Malondialdehyde (MDA) and superoxide dismutase (SOD) are important molecules involved in oxidative stress-induced damage. Our study showed higher MDA levels and reduced SOD activity in PCOS granulosa cells, confirming higher oxidative stress in these cells. Previous studies conducted in 2020 and 2024 on primary cultures of granulosa cells obtained from PCOS women also reported up to a threefold increase in intracellular ROS levels, confirming oxidative stress in these cells compared to the control group [38, 41].

In contrast, when we co-cultured PCOS-GCs with MenSCs, a significant decrease in the level of MDA and a considerable increase in SOD activity were detected. In addition, exposure of PCOS-GCs with MnsSCs-EXO significantly decreased the MDA level and nonsignificantly increased SOD activity. These results underscore the role of MenSCs and their exosomes in modulating oxidative stress. Chen et al. similarly investigated the antioxidant capacity of MenSCs and demonstrated their protective role against H2O2-related apoptosis in H9c2 cells, which enhances cell survival and migration [42].

Modulation of oxidative stress by MSCs and their derived exosomes is intricately linked with the improvement of mitochondrial biogenesis and related pathways. The master regulator of mitochondrial biogenesis, PGC-1α, plays a crucial role in regulating oxidative phosphorylation and the cellular response to oxidative stress [37, 43]. PGC-1α directly reduces oxidative stress by upregulating antioxidant enzymes and indirectly regulatesglucose metabolism, thereby mitigating ROS production [37, 44]. The study by Ying Liu demonstrated the pivotal role of PGC-1α in improving oxidative stress and cell survival of damaged granulosa cells [45].

Key pathways involved in activating mitochondrial biogenesis and PGC-1α include Akt, MAPK, and AMPK pathways. Pharmacological interventions and studies have targeted these pathways to exploit their therapeutic potential [37, 46]. MSCs and their derived exosomes have also been shown to activate these pathways, thereby enhancing mitochondrial biogenesis, and ameliorating oxidative stress [47, 48].

In PCOS, hormonal imbalance stands out as a prominent characteristic. Granulosa cells, pivotal for estrogen production in the ovaries, rely heavily on mitochondria, the primary site for steroidogenesis [49]. Mitochondria play a dual role in regulating steroidogenesis: controlling cholesterol, the steroid precursor, and managing the entry and processing of steroidogenic enzymes. This bidirectional relationship between mitochondrial structure and steroidogenesis impacts cells like granulosa, theca, and Leydig cells. Changes in steroidogenesis prompt alterations in mitochondrial structure and vice versa [50–52].

Studies on granulosa cells underscore the impact of mitochondrial dysfunction on steroidogenesis. PCOS model mice showed altered mitochondrial structure in granulosa cells [31]. Research on granulosa cells from individuals with endometriosis revealed reduced estradiol levels correlated with diminished mitochondrial mass and enzyme proteins, suggesting impaired steroidogenesis. These findings emphasize the critical role of mitochondrial function in steroid hormone production and its implications for reproductive health [49].

In our study, while direct examination of mitochondrial structure was not conducted, we demonstrated that mitochondrial dysfunction in PCOS granulosa cells was correlated with impaired estrogen production. Notably, we observed a significant reduction in estrogen production by granulosa cells in the PCOS-GCs group compared to the GCs group. Interestingly, following treatment with MenSCs and MenSCs-EXO, a substantial increase in estrogen production was observed in the treated groups compared to the PCOS-GCs group. These results underscore the role of MenSCs and their exosomes in enhancing estrogen production in PCOS granulosa cells by improving mitochondrial biogenesis [21, 53–55].

In our study, we found MenSCs to be more effective than MenSCs-EXO in improving abnormalities in PCOS granulosa cells. This superiority may be due to the continuous biological activity of live cells throughout the treatment period, as they actively secrete growth factors, cytokines, and microvesicles in response to their environment [56]. This results in a sustained secretion of MenSCs-derived factors with long-lasting effects on GCs. Conversely, in the MenSCs-EXO group, where only the initial dose was available, a short-term effect was observed due to the short half-life of exosomes, necessitating multiple doses for sustained impact [56]. Furthermore, during their active phase, cells produce various bioactive molecules in addition to exosomes [57], collectively exerting enhanced effects on GCs compared to the exosome-only group [58–61].

Despite these findings, there are several important reasons to continue researching exosomes derived from MenSCs. Firstly, exosome-based therapies offer significant practical advantages over cell-based therapies. Exosomes have lower tumorigenicity and immunogenicity, reducing the risk of adverse effects associated with live cell transplantation. They are easier to store and can be produced in large quantities, making them more suitable for clinical applications and large-scale treatments [62, 63]. Moreover, exosomes possess a natural ability to cross biological barriers, enhancing their potential for targeted delivery and systemic treatments. Additionally, while MenSCs can provide continuous secretion of bioactive molecules, using live cells can be challenging due to logistical and regulatory issues. Cell-free therapies, such as exosome-based treatments, on the other hand, avoid these challenges and offer a more controlled and standardized approach. Exosomes can be customized to carry specific therapeutic substances, potentially making them a more effective treatment option [59, 64].

In our study, co-culturing of MenSCs with PCOS granulosa cells led to the production of distinct and optimized secretions and exosomes compared to cells cultured under normal conditions. This indicates that MenSCs-EXO have significant therapeutic potential and should be further investigated. Although MenSCs were more effective than exosomes, the unique benefits of exosome-based therapies highlight the need for continued research in this area.

Future research should focus on improving exosome isolation, enhancing their stability, and exploring combination therapies using both exosomes and live cells. This approach could maximize therapeutic outcomes by combining the benefits of live cell activity with the practical advantages of exosomes, potentially leading to more effective treatments for PCOS and related conditions.

## Conclusion

Our study reveals the intricate relation between mitochondrial dysfunction, oxidative stress, and hormonal imbalance in the granulosa cells derived from PCOS model mice.

Our findings emphasize the crucial role of granulosa cells mitochondria in PCOS, with disruptions in mitochondrial biogenesis and function affecting estrogen production and oxidative stress levels. Additionally, we demonstrated the effectiveness of MenSCs and their exosomes in restoring mitochondrial biogenesis and reducing oxidative stress in PCOS model granulosa cells.

Further investigation into the mechanisms underlying the therapeutic effects of MenSCs and their exosomes is needed, along with clinical studies to validate their efficacy and safety in PCOS.

### Supplementary Information

Supplementary Material 1.

## Data Availability

The data used to support the finding of current study are available from the corresponding author, on reasonable request.

## Abbreviations

MenSCs: Menstrual Blood-Derived Stem Cells
PCOS: Polycystic Ovary Syndrome
GCs: Granulosa cells
FSH: Follicle-stimulating hormone
LHR: Luteinizing hormone receptor
POF: Premature ovarian failure
ER: Endoplasmic reticulum
MSCs: mesenchymal stem cells
PMSG: Pregnant Mare Serum Gonadotropin
FSHR: Follicle-stimulating hormone receptor
ICC: immunocytochemical
BCA: Bicinchoninic Acid
DLS: Dynamic Light Scattering
MDA: malondialdehyde
SOD: Superoxide dismutase
PGC-1α: Peroxisome proliferator-activated receptor gamma coactivator 1-alpha
NRF1: Nuclear respiratory factor 1
mtDNA: mitochondrial DNA
nDNA: nuclear DNA
IGF: insulin-like growth factor
POI: premature ovarian insufficiency
