# Supplementary material for: Granulosa cell insight: unraveling the potential of menstrual blood-derived stem cells and their exosomes on mitochondrial mechanisms in polycystic ovary syndrome (PCOS)
Source: J Ovarian Res. 2024 Aug 17;17:167. doi: 10.1186/s13048-024-01484-3 (PMC11330151; doi:10.1186/s13048-024-01484-3)
Supplement: Supplementary file 1 — Supplementary Material 1. [file 13048_2024_1484_MOESM1_ESM.pdf]

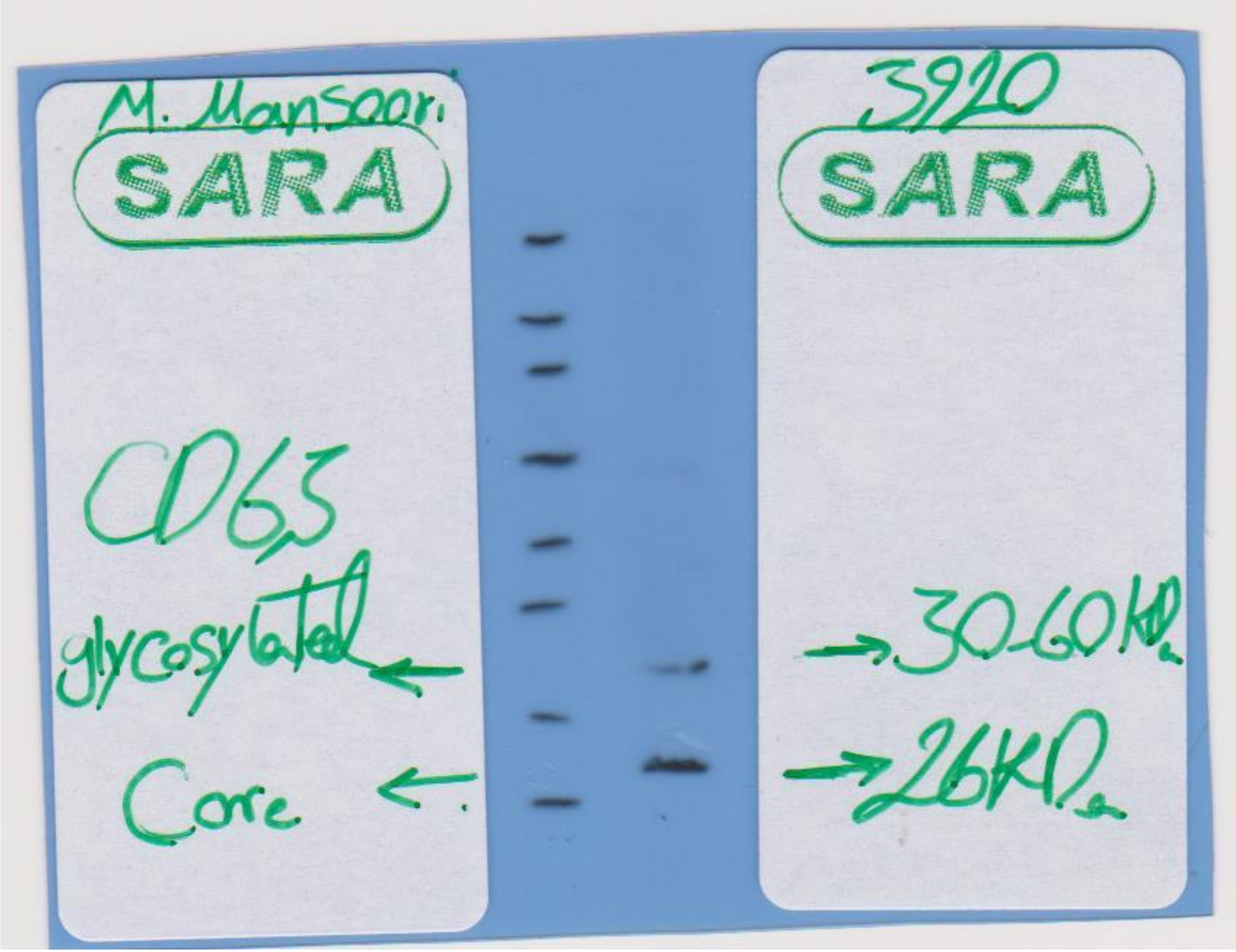
M. Mansoori
SARA
CD63
glycosylated
Core
3920
SARA
30-60 KDa
26 KDa

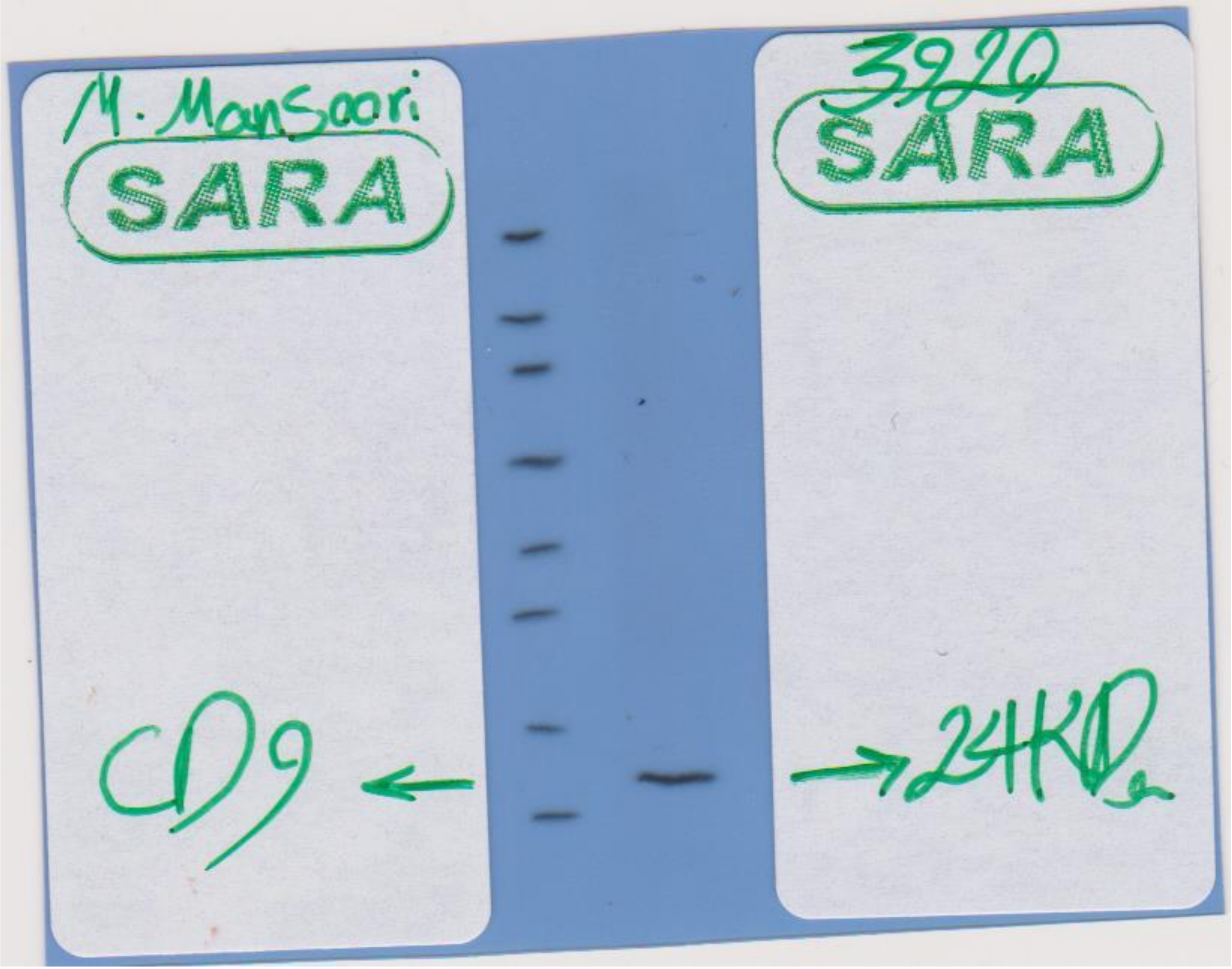
M. Mansoori
SARA
3920
SARA
CD9
24KDa

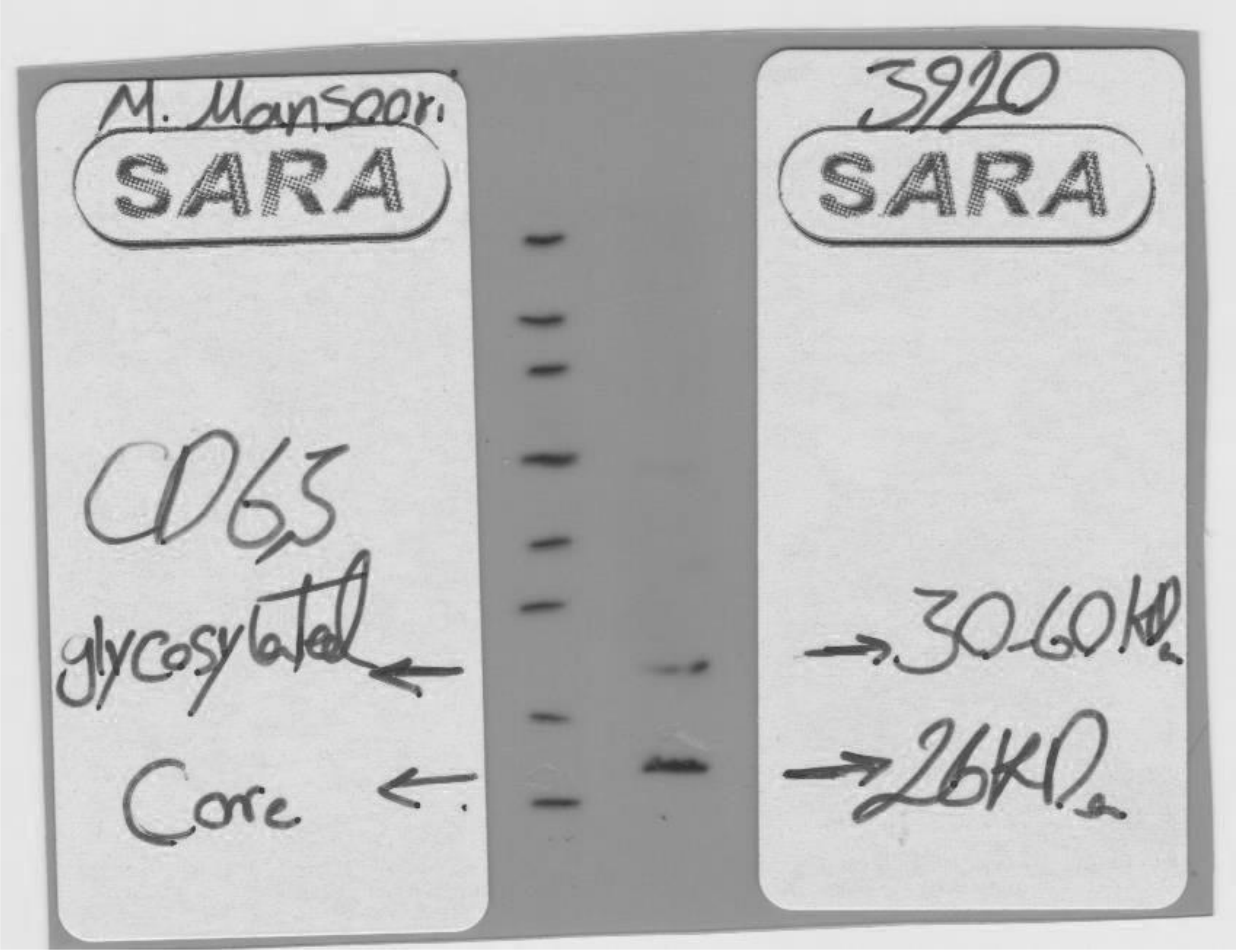
M. Mansoori
SARA
CD63
glycosylated
Core
3920
SARA
30-60KDa
26KDa

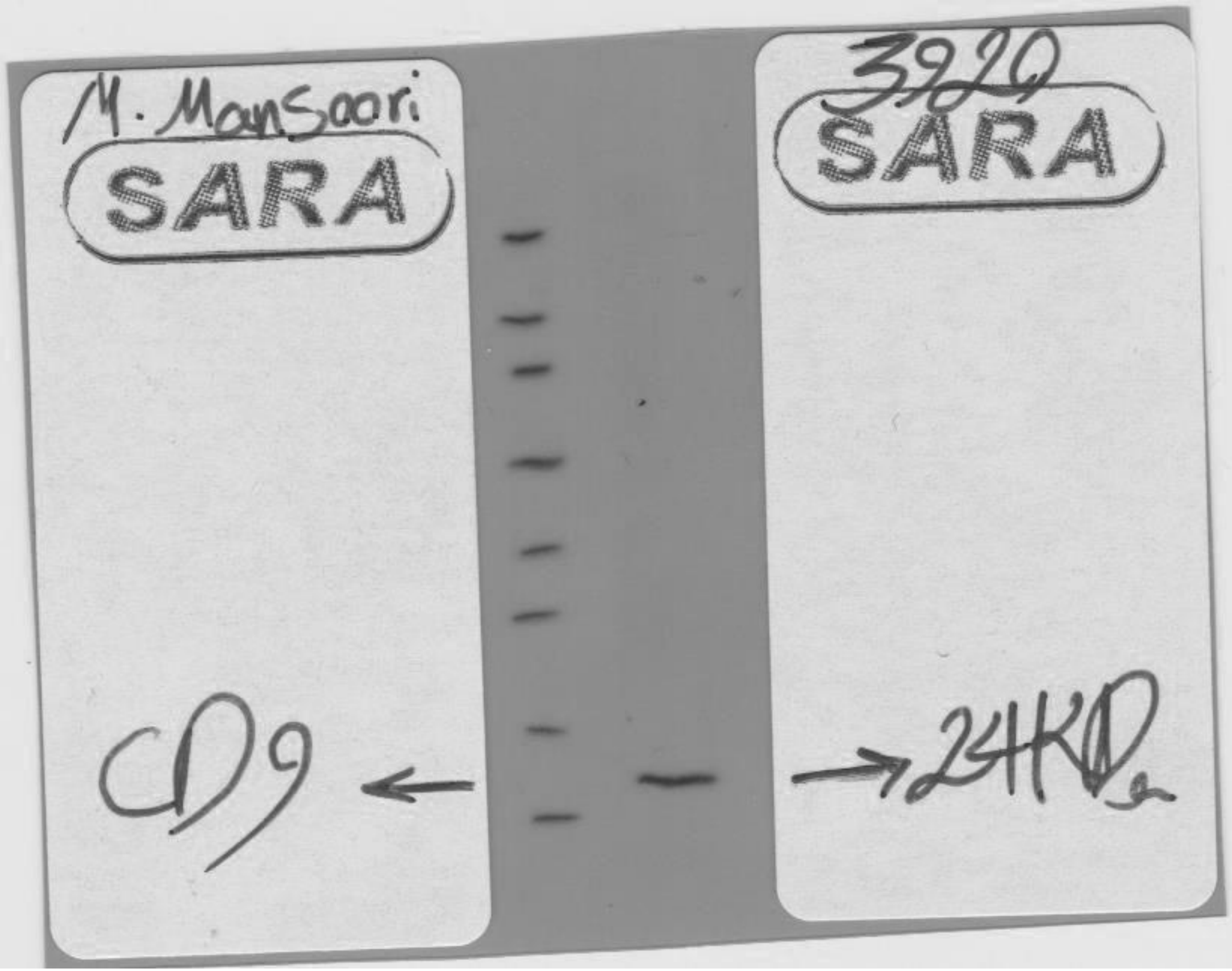
M. Mansoori
SARA
3920
SARA
CD9
24KDa
